# The mediating pathway linking social frailty and late-life malnutrition

**DOI:** 10.1093/geroni/igaf122.1091

**Published:** 2025-12-31

**Authors:** Doris S F Yu, Mary Miao, Chen Qiu

**Affiliations:** The University of Hong Kong, Hong Kong, China (People’s Republic); The University of Hong Kong, Hong Kong, China (People’s Republic); The University of Hong Kong, Hong Kong, China (People’s Republic)

## Abstract

**Background/Objectives:**

Social and mental health significantly influence the nutritional status of older adults. While a relationship exists between social frailty and malnutrition, the underlying mechanisms remain unclear. This study aims to explore the associations and potential psychological pathways among social frailty, depression, and malnutrition in Chinese older adults.

**Methods:**

A secondary analysis was conducted using data from the Jockey Club Pathway to Healthy Aging project, collected between May 2022 and June 2024. Structural equation modeling (SEM) was employed to examine the relationships among social frailty, depression, and malnutrition, as well as the mediating role of depression. Multi-group SEM was used to compare the hypothesized model across different BMI subgroups.

**Results:**

The study included 5,286 older adults, with a malnutrition prevalence of 0.6% and an at-risk malnutrition rate of 14.2%. The prevalence of social frailty and pre-frailty were 16.3% and 31.7%, respectively. Social frailty (β = -0.044, p = 0.004) and depression (β = -0.150, p < 0.001) were significant negative predictors of malnutrition scores, with depression mediating 55.1% of the total effect (β = -0.098, p < 0.001). Multi-group SEM revealed differences in path effects across BMI subgroups.

**Conclusions:**

The risk of malnutrition in older adults is influenced by social and psychological factors. Preventive strategies should differ based on BMI, focusing on social factors for overweight and obese individuals while addressing negative emotions in those with normal BMI to encourage healthy dietary habits and lifestyles.

